# A Novel and Secure Pseudovirus Reporter System Based Assay for Neutralizing and Enhancing Antibody Assay Against Marburg Virus

**DOI:** 10.3389/fmicb.2022.927122

**Published:** 2022-06-09

**Authors:** Jinhao Bi, Haojie Wang, Hongyan Pei, Qiuxue Han, Na Feng, Qi Wang, Xinyue Wang, Zhenshan Wang, Shimeng Wei, Liangpeng Ge, Meng Wu, Hao Liang, Songtao Yang, Feihu Yan, Yongkun Zhao, Xianzhu Xia

**Affiliations:** ^1^College of Veterinary Medicine, Jilin Agricultural University, Changchun, China; ^2^Changchun Veterinary Research Institute, Chinese Academy of Agricultural Sciences, Changchun, China; ^3^College of Animal Science and Veterinary Medicine, Henan Institute of Science and Technology, Xinxiang, China; ^4^College of Chinese Medicinal Materials, Jilin Agricultural University, Changchun, China; ^5^Institute of Laboratory Animal Science, Chinese Academy of Medical Sciences (CAMS) and Comparative Medicine Center, Peking Union Medical College (PUMC), Beijing, China; ^6^College of Animal Science and Technology, Shihezi University, Shihezi, China; ^7^College of Veterinary Medicine, Northeast Agricultural University, Harbin, China; ^8^Guangzhou Experimental Station, Chinese Academy of Tropical Agricultural Sciences, Guangzhou, China; ^9^Chongqing Academy of Animal Sciences, Chongqing, China

**Keywords:** Marburg virus, neutralization assay, pseudovirus, MR191, replicable pseudovirus

## Abstract

Marburg virus (MARV) is one of the principal members of the filovirus family, which can cause fatal hemorrhagic fever in humans. There are currently no prophylactic and therapeutic drugs on the market, and the high pathogenicity and infectivity of MARV make its research highly dependent on biosafety level 4 conditions, severely hindering the development of vaccines and therapies. Therefore, the development of medicines, such as MARV serological diagnosis, vaccines, and therapeutic antibody drugs, urgently needs a safe, convenient, and biosafety level 2 detection method to measure the neutralizing activity of MARV antibodies. To this end, we report a neutralization assay relying on a Rabies virus (RABV) reverse genetic operating system. We constructed infectious clones carrying the eGFP reporter gene and the full length of the original unmodified MARV GP gene. Based on the critical parameters of phylogenetic analysis, recombinant viruses targeting representative strains in the two major MARV lineages were successfully rescued. These pseudoviruses are safe in mice, and their inability to infect cells after being neutralized by antibodies can be visualized under a fluorescence microscope. We tested the system using the neutralizing antibody MR191. MR191 can significantly block the infection of BSR cells with pseudovirus. We compared it with the traditional lentivirus-type pseudovirus system to verify the system’s credibility and obtained the same results as reported in the literature. In general, we have established a safe and visualized method for evaluating the neutralizing activity of MARV antibodies. Compared with traditional methods, it has the advantages of convenient operation, short cycle, and low cost. It is a candidate method that can replace actual viruses for a neutralization assay.

## Introduction

Marburg Virus (MARV) is one of the principal members of the Filoviridae family, which can cause fatal hemorrhagic fever in human beings. Therefore, MARV and the Ebola virus (EBOV) have become the vital target of pan-filovirus research (Swenson et al., 2005; Keck et al., 2016; Bramble et al., 2018; Rahim et al., 2019). Unlike EBOV, MARV only includes Lake Victoria MARV and Ravn virus (RAVV; Barrette et al., 2011; Hume and Muhlberger, 2019). MARV can also be divided into Musoke, Angola, Pop, Ci67, and other strains according to the different locations of isolated strains and different isolates. The Ravn virus is named separately because of its far homology with other MARV (Cross et al., 2015; Mire et al., 2017).

Since it was first discovered in 1967, MARV has broken out many times (Ristanovic et al., 2020). The most severe outbreak of MARV occurred between 2004 and 2005, causing 252 cases and 227 deaths (Nyakarahuka et al., 2016). The fatality rate of this outbreak is as high as 90%. The root cause of the outbreak was the Angola strain. According to WHO data, the latest reported outbreak occurred on 6 August 2021, in a village in southern Guinea near the border of Sierra Leone and Liberia. One case was reported, and the patient died (2021). This is the first known confirmed case in West Africa (Mahase, 2021). MARV is mainly transmitted by blood, body fluid, and excreta. Most infected people were infected because of direct contact with infected wild animals, such as African green monkeys and fruit bats, or by handling their corpses and feces (Selvaraj et al., 2018; Paweska et al., 2020; Abir et al., 2022). MARV can cause the most severe viral hemorrhagic fever known to humans (Nyakarahuka et al., 2017; Siya et al., 2019). Unfortunately, there is no specific treatment or vaccine for Marburg hemorrhagic fever (MHF; Dulin et al., 2021). Therefore, it is urgent to develop vaccines and therapeutic drugs for prevention. In evaluating the immune effect of the vaccine, whether the body can produce a high titer neutralizing antibody after vaccination has always been a powerful indicator of humoral immune pathway evaluation (Klasse and Sattentau, 2002). It is generally believed that neutralizing antibodies induced by the vaccine can reduce the possibility of reinfection and the development of severe diseases, so identifying neutralization potency can reflect the relevance of immune protection (Bournazos et al., 2015). However, due to MARV’s high pathogenicity and widespread, scientific research must be carried out in the BSL-4 (biosafety level-4) laboratory. At the highest biosafety level, the accessibility of live virus staining has brought significant obstacles to developing candidate vaccines and treatment methods (Xiang et al., 2022). Although several companies have developed ELISA kits to detect specific antibodies to MARV, there is no information on the titer level of neutralizing antibodies to the virus. Therefore, it is urgent to develop an *in vitro* neutralization antibody detection method for MARV under the condition of BSL-2.

Glycoprotein is the only protein on the surface of MARV particles. That is a highly glycosylated type I transmembrane protein, which mediates the fusion of virus and host cells (Hashiguchi et al., 2015). That is an essential target for the MARV vaccine, antibody, and detection technology. MARV’s neutralizing antibody detection methods mostly use actual MARV and pseudovirus. The natural MARV method has high accuracy, but because MARV belongs to a grade 4 biosafety pathogen, it cannot be studied in a conventional BSL-2 laboratory. Because of its versatility and safety, the pseudoviruses reporting system has become an essential tool in virology research, especially for viruses with high pathogenicity and high biosafety (Muruato et al., 2020; Nie et al., 2020; Cao et al., 2021). Pseudoviruses are usually referred to as chimeric virus particles, which express the recombinant glycoprotein of another virus on the surface of a replication-deficient virus or virus vector. Pseudoviruses with replication defects can be used as an essential tool for virus detection, vaccine and drug research, and development (Li et al., 2018).

The neutralizing antibody detection method based on pseudovirus primarily uses HIV, VSV, and other vectors to package viral. From the perspective of biosafety, most of the packaging vectors used presently produce replication-deficient recombinant virus; that is, the virus particles obtained after packaging cannot be amplified *in vitro* and need to be “real-time rescue “brings experimental and economic pressure to relevant research, (Whitt, 2010; Vial et al., 2020; Salazar-Garcia et al., 2021). At the same time, a luciferase reporter gene is often used in the pseudovirus system. In detecting serum neutralizing antibody titer, the target cells infected by pseudovirus need to be lysed first and then interact with the substrate. After that, the instrument determines the relative light unit value to judge the neutralization of the sample to be detected with pseudovirus at each dilution. This neutralizing antibody detection method has many disadvantages, such as extended time, tedious steps, and high detection cost.

Based on the above questions, we report a novel and safe method to visualize MARV antibody neutralization activity, which can be operated at the BSL-2 level. Compared with traditional lentiviral pseudoviruses, it has a shorter experimental period and lowers economic costs. This method can detect the neutralizing activity against representative strains of two MARV lineages and is a relatively comprehensive evaluation system.

## Materials and Methods

### Biosafety and Ethics Statement

The recombinant virus and RABV SRV9 were studied in a Biosafety Level 2 Laboratory (BSL-2). All the experimental mice in this study were kept following the “National Standards for Welfare of Laboratory Animals in China” (GB14925-2010). This study was approved by the Animal Welfare and Ethics Committee of the Changchun Veterinary Institute of the Chinese Academy of Agricultural Sciences.

### Viruses, Cells, and Antibodies

The RABV SRV9 was cultured in adherent BHK-21 cells. Recombinant viruses were produced and cultured using the adherent BSR cell line (derived from BHK cells). 293 T cells were used to rescue lentivirus-type pseudoviruses and recombinant viruses. NIH3T3 cells were used to prepare the MARV cell line. The medium was high-glucose DMEM (C11995500CP, Gibco) and 5% fetal bovine serum (10,099,141, Gibco), and the culture conditions were 37°C and 5% CO2. Neutralizing antibodies were expressed in suspended 293F cells, and the culture medium used the basal medium OPM-293 CD05 (81075–001, OPM Bioscience) with complete chemical composition. The culture temperature was 37°C, the concentration of CO2 was 8%, and the speed was 125 rpm.

A fluorescein isothiocyanate (FITC)-conjugated anti-RABV-N monoclonal antibody (800–092) was purchased from Fujirebio, United States; Mouse anti-MARV Musoke strain (0203–023) and Angola strain (0203–025) specific monoclonal antibodies were purchased from IBT BIOSERVICES in the United States; Rabbit anti-MARV polyclonal antibody (ab190459), Horseradish Peroxidase (HRP)-conjugated mouse anti-human IgG Fc monoclonal antibody (ab99759), and Alexa Fluor 594-conjugated goat anti-rabbit antibody (ab150080) were purchased from Abcam, United States; PE-conjugated Mouse Anti-Human Lambda Light Chain antibody (MA1-10396) was purchased from Invitrogen, United States; Anti-mouse CD16/32 (101301) antibody were purchased from BioLegend, USA; HRP-conjugated goat anti-rabbit IgG antibody (ZJ2020), HRP-conjugated goat anti-mouse antibody (BS12478), and HRP-conjugated goat anti-human IgG antibody (BS10903) were purchased from Bioworld Technology, USA.

### Phylogenetic Analysis

The amino acid sequences of GPs of different strains of MARV published in GenBank were compared. Protein sequences included in the analysis: CAA82539 (Popp strain), ABA87127 (Musoke strain), ABE27085 (Durba strain), AAQ55258 (Ozolin strain), ABE27071 (Ravn strain), ABE27092 (Durba strain), ABE27078 (Durba strain), DQ447653 (Angola strain), ABS17558 (Ci67 strain), UFZ14320 (Latest reported strain). Evolutionary analyses were conducted in MEGA7. The evolutionary history was inferred using the neighbor-joining method and bootstrap test (1,000 replicates). Models with the lowest BIC scores (Bayesian Information Criterion) are considered to describe the substitution pattern the best. There were a total of 681 positions in the final dataset.

### cDNA Construction of Recombinant Virus

A recombinant virus carrying foreign genes was constructed using our previously described SRV9 strain RABV vaccine vector (Wang et al., 2015). The eGFP reporter gene was inserted into the vector through two restriction endonuclease sites, “BsiW I-Pme I.” The GP protein sequence of the MARV replaces RABV GP. According to the information published in GenBank, the glycoprotein sequences of MARV GP Angola strain (ID: KY047763.1), Musoke strain (ID: DQ217792.1), and Ravn strain (ID: KU179482.1) were inserted into the vector using the restriction enzyme cleavage site “Pst I-Kpn I.” Three full-length viral cDNAs expressing the eGFP reporter gene and MARV GP were constructed.

Full-length viral cDNA and helper plasmids pD-N, pD-P, pD-L, and pD-G (encoding individual structural proteins of RABV) were co-transfected into BSR cells using Lipofectamine 3,000 (L3000015, Invitrogen) transfection reagent as described previously. The fresh culture medium was replaced at 48 h and 96 h, respectively, and a fluorescence microscope was used for observation during the incubation period. The three recombinant viruses were named rSRV9-Angola-eGFP (rS-A), rSRV9-Musoke-eGFP (rS-M), and rSRV9-Ravn-eGFP (rS-R).

### TEM Analysis of Recombinant Virus

The morphology and size of the inactivated recombinant viruses were analyzed using Transmission electron microscopy (TEM) methods. In short, the virus solution and β-Propiolactone (33672.51, SERVA) were mixed in a ratio of 3,000:1 and hydrolyzed at 37°C for 2 h after standing at 4°C for 24 h. The inactivated virus solution and the copper-plated grids were fixed at room temperature for 60 min (mesh size was 200). The girds were removed, and a clean filter paper was used to absorb excess liquid from the grid’s edges. The grids were stained with 2% phosphotungstic acid solution (PTA) for 2 min at room temperature, after which excess liquid was removed. The grids were air-dried and observed using a transmission electron microscope (JEM-1200EXII, JEOL). TEM analysis was performed using an accelerating voltage of 80 keV and a magnification of 40,000×. The recombinant virus uses RABV as the vector and uses MARV GP instead of RABV GP. Therefore, under the transmission electron microscope, the recombinant virus should conform to the morphological characteristics of the *Rhabdoviridae* family. That is, the virus particles are bullet-shaped.

### Immunofluorescence Analysis

The BSR cells were seeded in a 12-well plate with cell crawling sheets in advance to infect the virus (MOI = 1). After 72 h, the cells were fixed with 4% paraformaldehyde for 1 h at room temperature. Block with 5% BSA solution overnight at 4°C. After 100-fold dilution with self-made rabbit serum of different strains, the cells were incubated at room temperature for 3 h. The Alexa Fluor 594-conjugated secondary antibody (ab150080) was then diluted 200-fold and incubated at room temperature for 1 h. The samples were encapsulated using an anti-fluorescence quencher (P0131, Beyotime) containing 4,6-diamidino-2-phenylindole (DAPI). The parental virus SRV9 was incubated with a mixture of rabbit sera against the three strains as a control. Immunofluorescence identification was performed using a confocal microscope model LSM800 from the manufacturer for ZEISS. Using a 63× oil lens (Plan-Apochromat 63×/1.40 Oil DIC M27, ZEISS) to observe the cell climbing sheets, the final magnification was 630×. Fluorescence observation uses 3 channels simultaneously. The AF594 dye used a filter cube (LSM800 GaAsP-Pmt2, ZEISS) with a 280 nm excitation filter and a 618 nm emission filter. eGFP fluorescence was detected using a filter cube (LSM800 GaAsP-Pmt1, ZEISS) with a 488 nm excitation filter and a 509 nm emission filter. DAPI dye was observed using a filter cube (LSM800 Airyscan, ZEISS) with a 353 nm excitation filter and a 465 nm emission filter.

### Western Blot

Viral fluid was collected, and the recombinant virus was inactivated using β-Propiolactone (33672.51, SERVA). Virus particles were precipitated with 1 M zinc acetate solution and resuspended in saturated EDTA solution 1/80 of the original virus volume. Centrifuge at 22,000 rpm for 90 min using a horizontal rotor at 4°C, and pass the resuspension through 20, 30, 40, and 55% sucrose solutions. Collect 30–40% and 40–55% white virus particles, centrifuge at 4°C, 30,000 rpm for 90 min, discard the supernatant, and dissolve the virus with an appropriate PBS solution. Purified viruses were denatured in a loading buffer at 70°C for 10 min. 20 μg of denatured protein was separated on a 4–20% SDS-PAGE gel. The separated proteins were transferred to PVDF membranes for western blot analysis. 5% skim milk solution was blocked overnight at 4°C. The 200-fold diluted MR191 antibody was incubated at room temperature for 3 h. This was followed by incubation for 1 h at room temperature using a 6,000-fold dilution of HRP-conjugated goat anti-human antibody (BS10903, Bioworld). Antibody responses of different strains adopt the following strategies. Mouse anti-Musoke-GP antibody (0203–023) and Angola GP antibody (0203–025) were from IBT. Ravn strain recombinant virus was incubated with rabbit anti-MARV polyclonal antibody (ab190459, Abcam). HRP-conjugated rabbit and mouse antibodies were diluted 90,000-fold and incubated at room temperature for 1 h. Bands were captured using a chemiluminescence imager after dropwise addition of electrochemiluminescence (ECL) immunoblotting substrate.

### Genetic Stability Analysis

The three recombinant viruses were serially passaged on BSR cells. The fifth and tenth generation viral fluids were collected, respectively. Reverse transcription was performed after extracting the viral genome according to the manufacturer’s instructions for TIANGEN (DP315). PCR identification and sequencing analysis were performed using the primers in Table 1 to confirm whether the viral genome was altered.

**Table 1:** Primers for sequencing validation of the MARV GP gene.

Primer name	Sequence(5′-3′)
Angola-F	ATGAAAACCACATGTCTCCTTATCA
Angola-R	TTATCCAATATATTTAGTAAAAATA
Musoke-F	ATGAAGACCACATGTTTCCTTATCA
Musoke-R	TTATCCGATATATTTAGTAAAGATA
Ravn-F	ATGAAGACCATATATTTTCTGATTA
Ravn-R	TCATCCAATGTATTTAGTGAAGATA

### One-Step Growth Curve

BSR cells were infected with the recombinant virus and RABV SRV9 at MOI = 0.1, and the supernatant was collected every 24 h to detect the virus titer. Samples to be tested were serially diluted ten-fold in serum-free DMEM. After the BSR cells were trypsinized, they were added to a 96-well plate at a final density of 1 × 10^6^ cells/ml. The ten-fold serially diluted virus solution was sequentially added to the well plate containing BSR cell suspension and cultured for 96 h. After fixation with pre-cooled 80% acetone solution at room temperature for 30 min, immunofluorescence staining was performed using FITC-conjugated anti-RABV N protein monoclonal antibody to determine the infection status of the virus.

### Safety Evaluation

Three-day-old ICR suckling mice were intra-cerebroventricular (IC) injected with virus solution (10^5^ TCID_50_), and the survival of mice was monitored every day for 30 consecutive days. Mice with central nervous system abnormalities, such as spasms and convulsions, will be euthanized. The suckling mice with central nervous system abnormalities, such as spasms and convulsions, were euthanized. Adult 8-week-old BALB/c mice were IC injection with virus solution (10^6^ TCID_50_), observed for 6 consecutive days, euthanized on the seventh day, and recorded the survival and body weight changes. The mouse brain tissue was taken for histopathological and immunohistochemical analysis on the seventh day.

### Design and Expression of Neutralizing Antibodies

According to the research information published by the two James E. Crowe (Flyak et al., 2015) and Erica Ollmann Saphire (Hashiguchi et al., 2015) teams, the variable region sequences of MARV MR191 (PDB: 6BP2) antibodies were synthesized. The signal peptide of IL-2 (GenBank: AAB86861.1) was added in front of the VH and VL genes of the antibody, and the modified pcDNA3.4 vector was added with the constant region of the antibody was inserted. The genetic design of the antibody can be viewed in Supplementary Material. Amino acid sequences were optimized for mammalian cells and synthesized by Sangon Biotech (Shanghai, China).

Two expression vectors (100 μg each) carrying antibody VH and VL genes were co-transfected into 200 ml 2 × 10^6^ cells/ml 293F cells using a cationic polymer transfection reagent (AC04L092, Life-iLab). The culture supernatant was collected after 7 days and passed through a 0.45 μm filter. The filtrate was passed through a Protein A affinity chromatography column (A4093101, SUNRESIN). Filler and wash impurities were equilibrated with a mixed solution of 20 mM PB and 150 mM NaCl (pH = 7.4). The targeting antibody was eluted with 100 mM sodium citrate solution (pH = 3.0). The antibodies were dialyzed in 0.01 M PBS (pH = 7.3) at 100 times the volume of the eluent at 4°C for 12 h. The purified antibody was passed through a molecular sieve of cross-linked dextran to remove multimers and stored at −20°C.

### Elisa

MARV-Angola, Musoke, Ravn GP1 genes were all added with the Strep purification tag (Tag: WSHPQFEKGGGSGGGGSGGSAWSHPQFEK) and inserted into the pCAGGS vector. Each expression vector (200 μg) was transiently transfected into 293F cells, and cell pellets were collected 7 days later. Use Binding buffer (100 Mm Tris-HCL, 150 mM NaCl, 1 mM EDTA, [pH = 8]) to resuspend the cell pellet, add protease inhibitor cocktail (M5293, AbMole), and sonicate the cells. After centrifugation, the supernatant was added with 11 U/mg avidin solution and incubated on ice for 30 min. The mixed solution was passed through a 0.45 μm filter and purified using a StrepTrap XT affinity chromatography column (29,401,328 AC, Cytiva) and a protein chromatography system (ÄKTA pure, GE).

The three proteins obtained after purification were suspended in coating buffer [50 mM Na_2_CO_3_, (pH = 9.6)] at a 0.5 μg/ml concentration, and 100 μl per well were inoculated into 96-well ELISA MaxiSorp plates. After overnight incubation at 4°C, the plate was washed three times for 5 min each with PBST (0.05% Tween-20 in 1 × PBS). Incubate at 37°C for 1 h with blocking buffer (1% BSA powder in 1 × PBST). MR191 antibody was serially diluted in blocking buffer (initial concentration 0.15 mg/ml) and incubated at 37°C for 2 h. After rewashing the plate, use a 1,000-fold diluted HRP-conjugated mouse anti-human IgG Fc monoclonal antibody (ab99759) to incubate at 37°C for 1 h. After washing with PBST, 100 μl per well tetramethylbenzidine (TMB, P0209, Beyotime) substrate was added. After 10 min, 50 μl of 2 M H_2_SO_4_ was added to each well to stop color development. The absorbance was read at 450 nm (OD450 nm) using a microplate reader (Multiskan Fc, Thermo Fisher).

### Construction of Cell Lines

The MARV-Angola/Musoke/Ravn GP gene sequence was inserted into the pCDH-CMV-MCS-EF1-puro vector. The signal peptide (SP) of MARV GP was replaced by the Murine Igκ-chain leader sequence (ID: AB050082.1, [9-71 bp]), which can target the protein to the secretory pathway; This is followed by the Hemagglutinin A epitope tag (Tag: YPYDVPDYA) which allows detection of the fusion protein by monoclonal antibody 12CA5; The transmembrane domain of MARV GP was replaced by Platelet-derived growth factor receptor transmembrane domain sequence [ID: DQ289579.1, (2049-2,598 bp)]. That allows the fusion protein to be anchored to the plasma membrane for display. The exogenous gene was inserted into the vector through two restriction endonuclease sites “EcoR I-Not I,” named pLV-MARV-A, pLV-MARV-M, and pLV-MARV-R, respectively.

**Figure 1:**
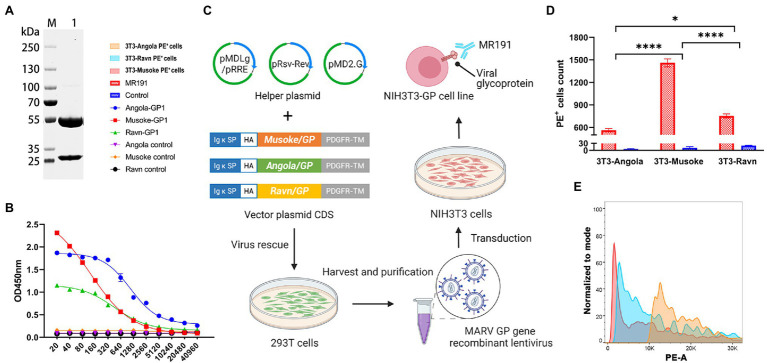
Binding activity of neutralizing antibody MR191 **(A)** SDS-PAGE identification of MR191. The reduced MR191 was identified using a 4–20% SDS-PAGE gel. The light chain can be detected at 25 kDa and the heavy chain at 55 kDa. **(B)** ELISA compared the binding ability of MR191 to GP1 of each strain. **(C)** Construction process of MARV GP-expressing 3 T3 cell line. Murine Igκ-chain leader sequence and HA sequence replace the original signal peptide of MARV GP. The original transmembrane domain was replaced by a Platelet-derived growth factor receptor transmembrane domain sequence to complete the mammalian cell display of GP. The engineered MARV GP was transduced by lentivirus to obtain cell lines. **(D)** Validation of the binding ability of MR191 to GP using cell lines. Under the premise of considering the influence of the control group, the number of PE cells in each group of cell lines combined with MR191 was used as an indicator for 2way ANOVA analysis to compare the differences in GP binding between MR191 different strains *in vitro*. The 3 T3-Musoke group was significantly higher than the other two groups (*p* < 0.0001). 3 T3-Ravn was higher than 3 T3-Angola (*p* = 0.0229). **(E)** PE^+^ signal in flow cytometry analysis after MR191 binding to three cell lines.

Using Liposome 3,000 Transfection Reagent (Invitrogen, L3000015), 10 μg pLV-MARV-A or 10 μg pLV-MARV-M, or 10 μg pLV-MARV-R were mixed with helper plasmids pMDLg/pRRE (7.5 μg), pRsv-Rev (3.75 μg), pMD2.G (4.5 μg) were co-transfected into 293 T cells (100 mm Dish). The supernatants were collected at 48 h and 72 h after transfection, and cell debris was removed at 3,000 × g for 15 min at 4°C. Viral fluid was concentrated according to the manufacturer’s instructions (EMB810A, ExCell Bio). Resuspend virus particles in 1/100 of the original volume of preservation solution (2.5% HEPES Buffer [15,630,080, Gibco] in DMEM [C11995500CP, Gibco]) and stored at −70°C. The three recombinant lentiviruses were named: LV-A, LV-M, and LV-R. According to the manufacturer’s instructions, three recombinant lentivirus titers were detected using the qPCR method (FV201, Transgen). NIH3T3 cells were transduced with 1/2 volume of virus solution with MOI = 40 at 37°C and 5% CO2 for 4 h and then filled up to the culture volume. After 24 h, the medium was replaced with a fresh medium to continue the culture. After 48 h, the fresh medium containing 9 μg/ml of Puromycin was replaced to continue the culture. The fresh medium containing puromycin was replaced every 2 days, and the monoclonal cell line was obtained by dilution after ten generations of continuous selection. The three cell lines were named: 3 T3-A, 3 T3-M, and 3 T3-R (Figure 1A).

### Flow Cytometry

Cell lines were scraped using cell Scrapers (FSCP023, Beyotime). The cell lines were stained with anti-mouse CD16/32 (101,301, BioLegend) antibody and incubated at 4°C for 10 min. Serial dilutions of MR191 were incubated with 3 T3-A, 3 T3-M, and 3 T3-R for 1 h at room temperature, respectively. The excess antibody was washed with sterile PBS. MR191 was incubated with PE-conjugated Mouse Anti-Human Lambda Light Chain antibody (MA1-10396, Invitrogen) for 1 h. After washing, fluorescence signals were collected using a flow cytometer (BD FACSVerse).

### Neutralizing Antibody Assay

#### Based on Lentiviral Vectors

The exogenous gene was inserted into the pcDNA3.1 vector through the two restriction enzyme recognition sites of “EcoR I-Not I,” named pcDNA3.1-A, pcDNA3.1-M, and pcDNA3.1-R, respectively. 293 T cells with a density of 6 × 10^5^cells/ml were seeded in 6-well plates and cultured for 12 h. 3 μg of donor plasmid (pcDNA3.1-MARV) and 3 μg of backbone plasmid (pNL4-3.luc.RE) were co-transfected into 293 T cells using lipofectamine 3,000 transfection reagent (L3000015, Invitrogen). The pseudovirus-containing supernatant was harvested after 48 h. 100 μl of diluted MR191 was incubated with 50 μl of virus solution at 37°C for 2 h, and then 50 μl of 4 × 10^5^ cells/ml 293 T cells were added. Cell control and virus control were set at the same time. The procedure was carried out in 96-Well White Opaque Plates (FCP968, Beyotime). After culturing for 48 h at 37°C with 5% CO2, discard 100 μl of the supernatant per well, add 100 μl of firefly luciferase substrate (RG055M, Beyotime) and incubate at room temperature for 5 min, and then use a multi-plate reader for chemiluminescence detection (SYNERGY H1, BioTek). Calculate the neutralizing activity of the sample based on the luciferase activity: neutralizing activity (%) = [(virus control RLU - sample RLU)/(virus control RLU - cell control RLU)] × 100%. The well at which the luciferase activity decreased by more than 50% was considered the neutralizing activity.

#### Based on RABV SRV9 Vectors Serial Fold Dilutions of MR191

**Figure 2:**
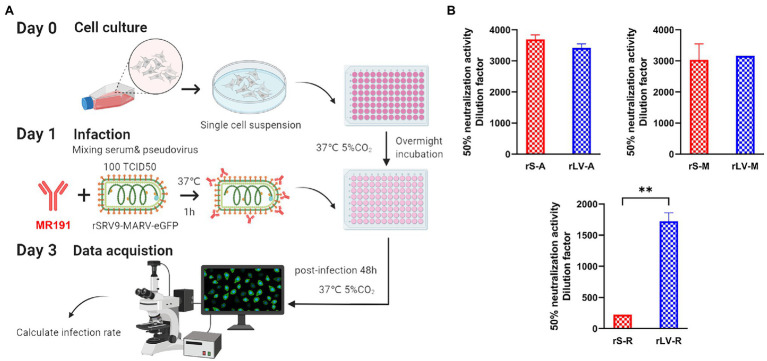
Pseudovirus neutralization assay based on SRV9 reverse genetic operating system **(A)** Neutralization assay operation process. The pseudovirus of 10^2^ TCID_50_ was incubated with the test sample (such as serum) with or without nAb at 37°C for 1 h. The incubated mixture was inoculated into BSR cells and continued to incubate for 48 h, followed by fluorescence microscopy to assess the samples’ neutralizing activity. If nAb is present in the sample or the antibody has neutralizing activity, the pseudovirus is neutralized, and no green fluorescence in the form of flaky aggregates can be observed after 48 h incubation of the mixture. Conversely, a large amount of green fluorescence can be observed. **(B)** Comparison results with lentivirus-type pseudovirus systems. Data represent the mean ± Standard error of the mean (SEM) of 3 replicate tests per sample. For normalization of the results, the infectious dose that protects 50% of the cells from fluorescence is calculated by the Reed–Muench method. The calculated dilution factor is used as the neutralization potency. Differences between the two groups of data were analyzed by *t*-test.

Take 100 μl of each dilution sample and mix it with 10^2^ TCID_50_ recombinant viruses (rS-A, rS-M, rS-R). After incubation at 37°C for 2 h, 50 μl of 1 × 10^6^ cells/ml BSR cells were added, and cell control and virus control were set. The results were observed under a fluorescence microscope after culturing for 48 h. Using the Reed–Muench method, the dilution of the sample that can protect 50% of the cell wells from producing fluorescence was calculated, and the dilution was the neutralization titer of the sample (Figure 2A).

### Data Processing

Flow cytometric analyses were performed using FlowJo software (version 10.7.2). All data represent the mean ± SEM. Comparisons of 2 groups were analyzed using an unpaired, 2-tailed *t*-test. Comparisons of 3 or more groups were analyzed using ANOVA. A *p*-value of less than 0.05 was considered statistically significant. Analyses were performed using GraphPad Prism (version 9.02).

## Results

### Sequences Analysis

**Figure 3:**
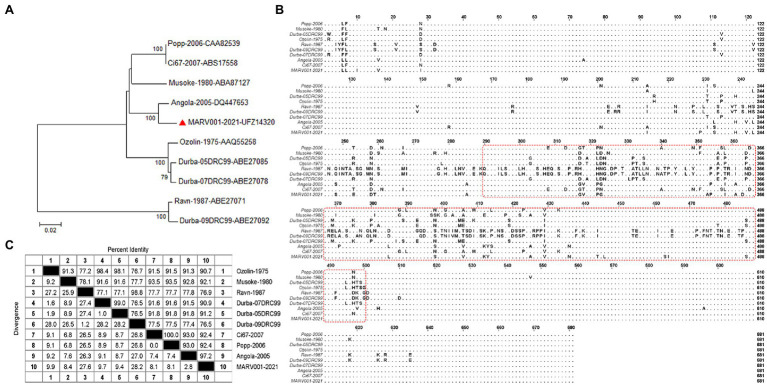
Phylogenetic analysis of representative strains of MARV **(A)** The amino acid sequence of MARV GP in the GenBank database was analyzed using MEGA (version 7.0.20). The Jones–Taylor–Thornton (JTT) model was the optimal amino acid substitution model. Phylogenetic analysis used a neighbor-joining tree and 1,000-repeat bootstrapping. The ruler at the bottom of the dendrogram indicates the number of nucleotide substitutions per site. Horizontal branch lengths are proportional to the genetic distance between sequences. Individual strains consist of the name and GenBank accession number, and the numbers on the left branch are percentages of bootstrap values. The red triangles represented the most recently discovered strains in 2021. **(B)** The figure is a multiple sequence alignment of GP amino acids for representative strains in **(A)**. MARV GP is mainly composed of GP1, Mucin, and GP2. The amino acid differences are mainly concentrated in the Mucin part. The red box outlines the amino acid sequence (aa290-500) within the mucin-like domain. **(C)** Analysis of amino acid sequences among MARV strains. That shows the homology of GP amino acids among different strains. The upper half of the dividing line represents the homology of GP amino acid sequences between MARVs, and the lower part represents the differences. The numbers correspond to different strains.

Phylogenetic analysis showed that MARV strains were divided into two major branches. One of these branches includes the Ravn strain, isolated in Kenya in 1987, and the Durba strain (DRC1999 strain), discovered in the Democratic Republic of Congo in 1999. The other central branch is again divided into two branches. One of the subclades consists of three strains represented by the Ozolin strain isolated in 1975 (05DRC99 and 07DRC99). Another subclade consists of Popp, Ci67, Musoke, Angola, and MARV001. The MARV001 was newly isolated in August Guinea tMoHo (2021). The close relationship between the Angola and Musoke strains may explain the phenomenon that many MARV vaccines using either as an antigen can produce cross-protection between the two. That may make the probability of cross-protection in the more distantly related Ravn strains minimal (Figure 3A). We compared the GP amino acid sequences of MARV isolates. We found that the amino acid differences among the strains were mainly concentrated near the mucin-like domain (aa290-500; Figure 3B), which resulted in a massive span of 76.5–99% amino acid homology between different lineages (Figure 3C). In filoviruses, the spatial structure of the mucin domain may cause differences in the binding of monoclonal antibodies to EBOV and MARV, a feature that may be reflected in MARV antibodies cross-reacting with EBOV. Therefore, the amino acid diversity of MARV mucin domains is reflected in the phylogenetic branches of each isolate and may also suggest the binding characteristics of antibodies to pan-filoviruses. That makes it more critical to develop an assay for neutralizing MARVs of different lineages.

Based on the above phylogenetic analysis results, the closer genetic relationship between Angola and Musoke suggests that neutralizing antibodies against Ravn strains may not be recognized by the first two types of pseudoviruses. Although in some viruses with highly conserved genetic evolution, a single strain can produce cross-active neutralizing antibodies. It is worth noting that there are often many differences in the etiological characteristics and clinical symptoms between different strains. Therefore, developing a neutralizing antibody test against a virus needs to cover at least all significant lineages rather than just a specific strain. To this end, we developed MARV pseudoviruses against three strains of Angola, Musoke, and Ravn.

### Expression of MARV GP and eGFP by Recombinant Virus

**Figure 4:**
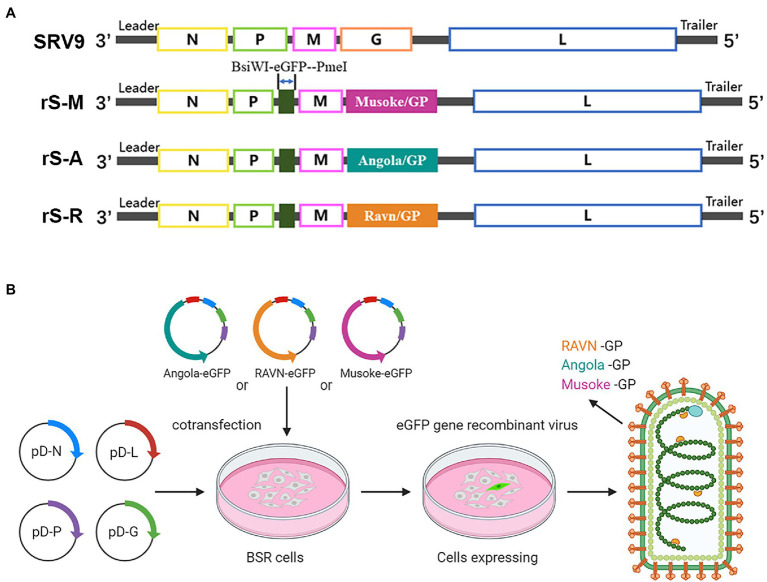
Construction process of recombinant virus **(A)** The eGFP reporter gene was inserted between the complete genomic sequences encoding RABV phosphorylated protein (P) and matrix protein (M) through two sites, “BsiW I-Pme I.” The original glycoprotein gene sequence of RABV was replaced with the GP sequence of the corresponding strain of MARV to complete the genome modification of the recombinant virus. **(B)** The full-length vector carrying the modified complete genome was transfected into BSR cells together with the helper vector expressing the four structural proteins of RABV to complete the packaging of the recombinant virus. Three recombinant viruses with rhabdovirus morphology and green fluorescence properties were obtained, and the surface glycoprotein was MARV GP.

MARV GP is a highly glycosylated envelope glycoprotein. To restore the natural structure of MARV GP, we did not make any additional modifications to GP (including codon optimization). However, since the original sequence of MARV-Angola is complex and can be recognized by a large number of restriction endonucleases, to make better use of the polyclonal restriction site reserved for the RABV SRV9 vaccine vector, we ensure that the amino acid remains unchanged. MARV-Angola GP 560-561 bp “CGT” was replaced with “AGG.” That allows the use of a “Pst I” site at the 5′ end of the three GP full-length cDNAs and a “Kpn I” site at the 3′ end for easy manipulation (Figure 4A). For visualization, we cloned the eGFP reporter gene to flank the RABV transcription start/stop signal located between the P and M genes, using two unique restriction endonuclease sites “BsiW I-Pme I” accomplish.

**Figure 5:**
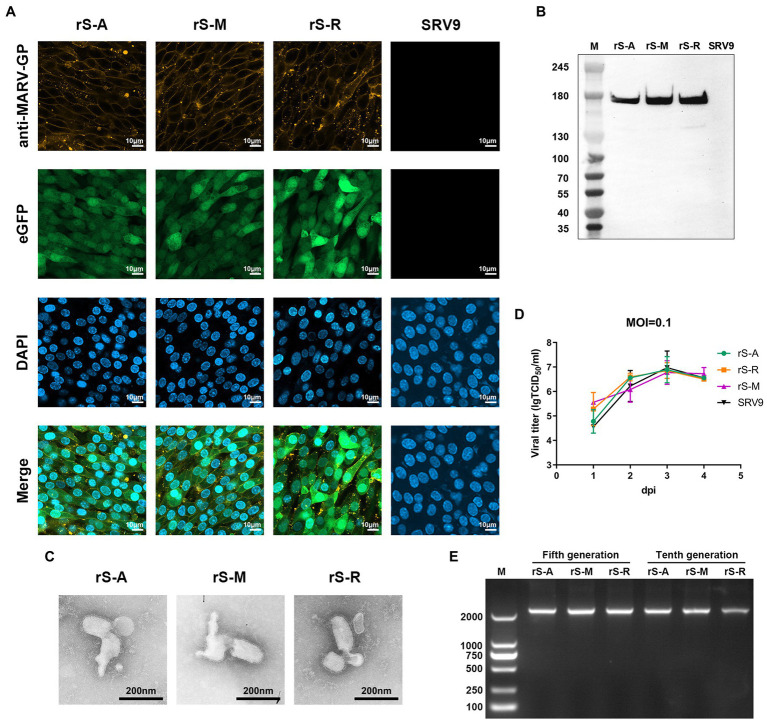
Properties of pseudoviruses **(A)** Confocal microscopy analysis of BHK cells infected with the recombinant virus at MOI = 1 for 72 h. BHK cells were not permeabilized. The expression of the eGFP reporter gene was verified using excitation light with a wavelength of 488 nm. According to the budding process of RABV, the infected cells can express MARV GP. Therefore, the recombinant virus was analyzed using the rabbit serum (1:100) corresponding to the strain and the AF594-conjugated secondary antibody (1:200), respectively, under the channel of excitation light wavelength of 590 nm. Nuclei were labeled with DAPI. **(B)** Western blot identification of the recombinant virus. Using a 200-fold dilution of MR191 to incubate with PVDF membrane, the expression of MARV GP by the recombinant virus was verified at the protein level. **(C)** The proper morphology of the pseudovirus under transmission electron microscopy. The scale bar is 200 nm. **(D)** One-step growth curves of three pseudoviruses compared to the parent virus. After the parent and recombinant viruses were inoculated into BSR cells at MOI = 0.1, the virus titers were detected from the first day to the fourth day. In 2-way ANOVA analysis, the titer of rS-M at 1dpi was slightly higher than that of other viruses, and there was a difference compared with SRV9 *p* = 0.0135. Other viruses were not different from SRV (*p* > 0.05). **(E)** RT-PCR identified the stability of the MARV GP gene insertion recombinant virus.

Infectious cloned full-length vectors representing three strains were co-transfected into BSR cells with four helper vectors to rescue the recombinant virus (Figure 4B). All three recombinant viruses appeared with green fluorescence 96 h after transfection, and all were successfully recovered. BSR cells were infected with MOI = 1 to verify the expression of foreign proteins. Immunostaining was performed with rabbit hyperimmune sera against different strains and Alexa Fluor 594-conjugated secondary antibodies. In the absence of cell permeabilization, orange fluorescence from Alexa Fluor 594 fluorescent dye can be observed at the edge of the outline of cells infected with recombinant virus (Figure 5A). That indicates the presence of MARV GP on the surface of cells infected with the recombinant virus.

In order to detect foreign proteins introduced by the recombinant virus, immunoblotting (WB) was used to analyze inactive sucrose-purified virions after infection of BSR cells. The results showed that the target band of GP1 could be detected at 180 kDa using MR191 as the specific antibody (Figure 5B). The three recombinant viruses use their respective specific antibodies (Supplementary Data 2). rS-A was detected using mAb (0203–025) expressing recombinant MARV of Angola GP as immunogen. mAb 5C1 (0203–023) reacts specifically against MARV-Musoke glycoprotein. It does not react against Ravn GP, or the Angola GP. rS-R was analyzed using a rabbit polyclonal antibody whose immunogen was a synthetic peptide and could recognize the unique sequence of the MARV GP2 subunit. The results showed that both rS-A and rS-M could detect the target bands around 180 kDa and 45 kDa, which were consistent with the protein sizes of GP1 (170 kDa) and GP2 (40 kDa) reported in the literature. According to the manufacturer’s description for the antibody (ab190459), rS-R detects the specific MARV GP2 target band (74 kDa) around 70 kDa. The specific synthetic peptide from which this antibody was made was 35–40 kDa for Musoke GP and around 30 kDa for Angola GP. Therefore, this antibody was used to recognize Ravn GP specifically. A phenomenon was discovered during the analysis of WB. MARV GP is highly glycosylated and often detects at a molecular weight higher than the theoretical size due to the altered mobility. This phenomenon is consistent with the results reported on MARV WB analysis (Keshwara et al., 2019).

The morphology of the recombinant virus was observed by electron microscopy to confirm whether the presence of foreign proteins affected the structure of the virus particle. The results showed that the morphological characteristics of the three recombinant viruses were consistent with the morphological structure of rhabdoviruses (Figure 5C).

These results further confirmed that the insertion of eGFP and GP could successfully package the recombinant virus with rhabdovirus morphology after removing RABV GP. Virus-infected BSR cells have green fluorescence properties. MARV GP can be correctly expressed, folded, and transported to the cell surface by the RABV SRV9 vector, following the budding characteristics of members of the *Rhabdoviridae* family. Probed of GP1 and GP2 at the protein level also confirmed that GP can complete cleavage and processing after integration into virions. In conclusion, we constructed three MARV pseudoviruses, rS-A, rS-M, and rS-R, which cover the two major lineages of MARV.

### Genetic Stability and Growth Characteristics

To explore the difference in growth kinetics between each recombinant virus and the parent virus, we uniformly selected the 5th generation virus culture supernatant, inoculated BSR cells at a dose of MOI = 0.1, collected the supernatant for 4 consecutive days, and determined the titer. The positive cells infected with the recombinant virus were observed by fluorescence microscope, and the parental virus was determined after immunofluorescence staining with FITC-conjugated anti-RABV-N mAb. The results showed that the titer of rS-M at 1dpi was slightly higher than that of other viruses. The growth characteristics of the other two pseudoviruses did not show statistical differences from the parental virus. Overall, the growth trend of the three recombinant viruses was consistent with that of SRV9, reaching the maximum value at 3dpi and then showing a downward trend (Figure 5D).

In order to verify whether the MARV GP gene was stably expressed during the passage of each recombinant virus, viral genomes were extracted from the culture supernatants of the fifth passage (F5) and tenth passage (F10) of infected BSR cells. RT-PCR was performed using each strain’s GP sequence primers (Table 1) and verified by sequencing (Supplementary Data 3, 4). The results showed that specific nucleic acid bands could be detected in the three recombinant viruses, F5 and F10 (Figure 5E). The sequencing results showed that Angola GP, Musoke GP, and Ravn GP could be stably inherited in the recombinant viruses.

These results based on replication and spread of viruses with recombinant genomes suggest that the kinetic growth properties of recombinant viruses are not affected by exogenous proteins. The growth trend of recombinant viruses was highly similar to that of the parental virus at MOI = 0.1 and peaked at 3dpi. In addition, the MARV GP gene can be stably maintained and expressed.

### Pathogenicity in Mice

**Figure 6:**
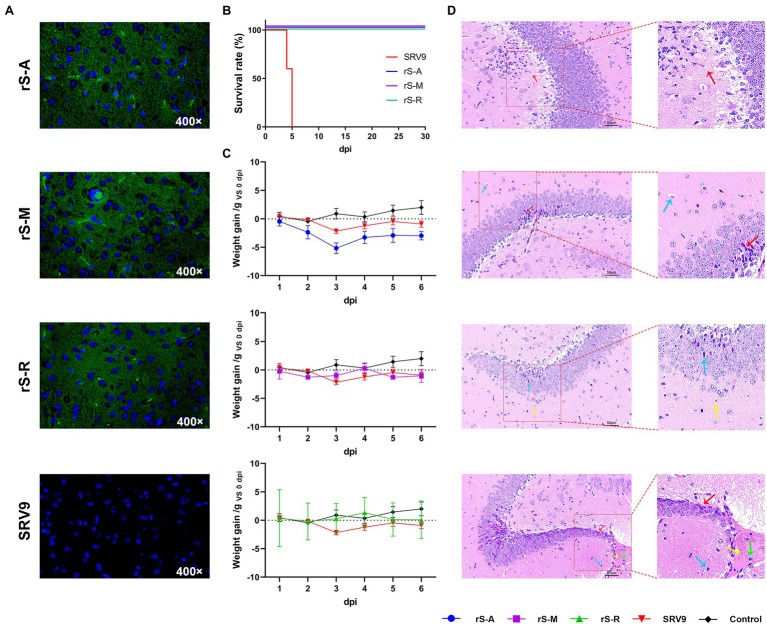
Safety Evaluation of Pseudovirus **(A)** After intracranial inoculation of pseudovirus in adult mice, the proliferation and replication of the virus in the mouse brain were observed by fluorescence microscope. Pseudoviruses carrying fluorescent properties can infect the brain tissue of adult mice and replicate and proliferate generally in the brain. Green fluorescence can be directly observed in brain slices under a fluorescence microscope, but the parental virus does not possess this property. **(B)** Survival curve of 3-day-old ICR suckling mice after intracranial inoculation with pseudovirus. The parental virus SRV9 group (n = 5) started to die on the fourth day, and the survival rate decreased to 0% on the fifth day. The three pseudoviruses obtained by manipulating the parental virus showed high safety, which is reflected in the fact that under the same conditions, all the suckling mice inoculated with the three pseudoviruses survived until the end of the experimental observation period (30 days). **(C)** Bodyweight changes after intracranial inoculation of pseudovirus in adult mice. The figure shows the change in the experimental and control groups’ body weight during the observation period after inoculation compared with that before inoculation. All experimental group data were compared with the PBS control group, with 0 as the fluctuating baseline (dashed line), and there was a significant difference between rS-A and PBS (*p* = 0.0032). **(D)** Histopathological analysis of adult mouse brain after inoculation with pseudovirus. Adult mice were randomly selected after intracranial inoculation with pseudovirus on the seventh day. The brain tissue was fixed with 4% paraformaldehyde, and the vicinity of the hippocampal formation was selected for analysis. Arrows indicate local lesions in sections of brain samples, and the color of the arrows distinguishes the different pathological changes shown within each sample.

Although MARV was first identified in Germany, most reported cases are in African countries, including Guinea. However, natural MARV needs to be operated in a biosafety level 4 laboratory. That limits the evaluation of neutralizing activity of antibodies. On the premise that three pseudoviruses can complete replication and proliferation in the murine brain (Figure 6A), we performed a pathogenicity evaluation in mice to develop a safe method to be tested in a level 2 laboratory.

3-day-old ICR suckling mice were intracranially inoculated with viruses (10^5^ TCID_50_) and observed for 30 days. Mice inoculated with the parental virus showed lethargy on the second day, reduced limb swings, dead individuals on the fourth day, hunched back and limb convulsions on the fifth day, and all died within the same day. In contrast, all mice inoculated with the pseudovirus survived the observation period (Figure 6B).

SRV9 is an attenuated vaccine strain obtained by plaque-cloning a BHK cell-adapted SAD strain and therefore does not pose a lethal threat to adult mice. In adult mice intracranially inoculated with viruses (10^6^ TCID_50_), we monitored clinical responses (including changes in body weight and abnormal behavior) and performed a histopathological analysis of the brain. After inoculation of SRV9 in adult mice, the bodyweight change from 3dpi was lower than the original level and declined (Figure 6C). Brain pathological analysis showed the tissue structure was severely abnormal, and a large number of neuronal degeneration and hyperchromatic cells were seen in the hippocampus (red arrows); Large areas of neuronal coagulation-like necrosis are seen in the tissue (yellow arrows); Individual inflammatory cells can be seen in the necrotic area (green arrows); Small triangular basophils (blue arrows) are visible at the edge of the necrotic area (Figure 6D).

The rS-A group showed decreased activity at 2dpi and the food intake during the observation period was significantly lower than all groups. Weight change is in a downward trend, with 3dpi dropping the most and then picking up (Figure 4C). Brain pathological analysis showed that the tissue structure was mildly abnormal, and a small amount of inflammatory cell infiltration (red arrow) was seen in the tissue; the neurons in the hippocampus showed no degeneration, and the cell structure was clear and neatly arranged (Figure 6D).

The bodyweight of the rS-M group fluctuated slightly after 1dpi and 4dpi, respectively. During the observation period, the mice’s food intake and water intake in this group were slightly higher than those in the rS-A group. The nocturnal activity was significantly reduced at 2dpi (Figure 6C). The brain tissue structure was mildly abnormal, with a small amount of neuronal degeneration in the hippocampus (red arrow); the neuronal structure in the hippocampus was clear and neatly arranged, and no other lesions were seen; HE staining showed microvessels (blue arrow; Figure 6D).

The rS-R group showed less fluctuation in overall body weight, and no abnormal behavior was observed (Figure 6C). The structure of brain tissue was slightly abnormal, with a small amount of neuronal degeneration in the hippocampus, and cells were deeply stained (blue arrow); HE staining showed microvessels (yellow arrow); the nuclei of the cells were clear, and no obvious necrosis was found; the cells in the tissue were tightly arranged without edema (Figure 6D).

The three pseudoviruses showed excellent safety in suckling mice and were suitable for BSL-2 manipulation. Histopathological analysis showed that the modified three replicable pseudoviruses did not enhance the pathogenic effect on brain tissue compared with the parental virus. This phenomenon may be related to the replacement of RABV GP by MARV GP, or the larger genome after the modification indirectly affects the pathogenic effect of the recombinant virus.

### MR191 Binding and Neutralization Activity

It is essential to use standard neutralizing antibodies as a positive control to validate the evaluation method of neutralizing activity. To test whether recombinant viruses carrying the eGFP reporter gene could serve as a valid model for assessing antibody neutralizing activity, we expressed neutralizing antibody (MR191) isolated from survivors of MARV infection. SDS-PAGE results showed that MR191 obtained by the 293F expression system could simultaneously detect light chain and heavy chain, with typical IgG characteristics (Figure 1A). According to Flyak et al. (2015), MARV survivor neutralizing antibodies bind to a single antigenic site and bind to the receptor-binding site (RBS) region. MARV GP contains two subunits, GP1 and GP2, and RBS is located on GP1. Therefore, we evaluated the binding capacity of MR191 to Angola GP1, Musoke GP1, and Ravn GP1 and the intact GP of each strain. A 20-fold dilution of 3 mg/ml MR191 was used as the initial concentration, followed by serial doubling dilutions. ELISA results showed that LogIC50Musoke GP1(2.090)<LogIC50Ravn GP1(2.661)<LogIC50Angola GP1(3.034). That indicates that MR191 has more substantial binding specificity and higher sensitivity to Musoke GP1 protein (Figure 1B).

To confirm the binding ability of MR191 to MARV GP, we used the 3 T3 cell line displaying GP on the surface as a model and used flow cytometry to analyze (Figure 1C). MR191 was used as the primary antibody, and PE-conjugated anti-human lambda light chain was used as the secondary antibody to compare the binding ability of MR191 to cell lines expressing different GP. The order of binding ability from strong to weak was 3 T3-Musoke>3 T3-Ravn>3 T3-Angola (Figures 1D,E). This result is consistent with the ELISA result.

MR191 is currently the only neutralizing antibody with complete protection in non-human primates following MARV exposure (Mire et al., 2017). After binding to GP, all residues that MR191 contacts are highly conserved in MARV. No single variation in these residues has been found in any strain since the discovery of MARV (King et al., 2018), so we compared the binding activity of MR191. The results showed that MR191 could bind to the uncut intact GP and truncated GP1 of the three strains. The binding ability for each strain GP or GP1 was consistent, with the most substantial binding ability to Musoke and the weakest to Angola.

### Comparison of Neutralization Assay

The neutralizing activity of MR191 was evaluated using three pseudovirus systems (Figure 2A). The luciferase activity decreased by more than 50% as the criterion for the lentiviral pseudovirus system. Method Reed–Muench (Zamoiskii, 1956; Ramesh et al., 2020) analyzed the neutralization activity and reflected on the dilution factor. T-test was used to compare the difference in dilution factor that could neutralize more than 50% of pseudoviruses between the two methods. The results showed that there was a significant difference between the two pseudoviruses against Ravn strains; the difference between means (rLV/A-rS/A) ± SEM = −272.9 ± 193.3; compare rLV-M with rS-M, 131.8 ± 517.6; compare rLV-R with rS-R, 1498 ± 136.8. It can be seen from Figure 2B that the neutralization activity detected by the rLV-R method is about 1,500 times higher than that of rS-R. Both pseudoviruses were used for 10^2^ TCID_50_ during the experiment, but the two tests against the Ravn strain presented very different results (Figure 2B).

In the longitudinal comparison, we found that the neutralizing activity of MR191 against Angola and Musoke was significantly higher than that of Ravn in the detection results of recombinant pseudoviruses using rSRV9 (Figure 2B). The recombinant pseudovirus of the lentiviral vector showed the same difference as rSRV9. The initial concentration of MR191 was 3 mg/ml, and the lower limit of detection of MARV by rSRV9 pseudovirus system was Mean_(Angola)_ = 3692.45 (0.81 μg/ml); Mean_(Musoke)_ = 3030.51 (0.99 μg/ml); Mean_(Ravn)_ = 223.872 (13.40 μg/ml). The detection limit of MARV by the lentiviral pseudovirus system is Mean_(Angola)_ = 3419.52 (0.88 μg/ml); Mean_(Musoke)_ = 3162.28 (0.95 μg/ml); Mean_(Ravn)_ = 1721.68 (1.74 μg/ml), Concentration results are rounded to two decimal places. The neutralization activity of MR191 in different strains obtained by the two methods was the same, Angola>Musoke>Ravn.

## Discussion

During the genetic evolution of viruses, the unstable living environment creates different selection pressures, which allows the adaptive mutations of the virus to be retained and survive, and then develop into different genotypes and strains. In addition, after the virus infects host cells, to better avoid the attack of the humoral immune response, it often develops in the direction of generating escape mutants (Park et al., 2020; Tohma et al., 2022). So, when developing a vaccine against any virus, scientists have received significant attention to the overall protective effect and ability to prevent infection by different strains and isolates of the same virus. Although in filoviruses, MARV does not have multiple viral subtypes, unlike EBOV. A vaccine study based on the Venezuelan equine encephalitis virus (VEEV) replicon platform demonstrated that cynomolgus monkeys vaccinated with the Musoke strain were resistant to lethal homologous challenge (Hevey et al., 1998) but not to the survival challenge of the Ravn strain (Hevey et al., 2001). GP is the only glycoprotein on the surface of filoviruses, which mediates the attachment and entry of virions into target cells. It is an important target for the development of MARV therapeutic antibodies and vaccines. Our phylogenetic analysis of MARV GP found that Ravn GP differed from another lineage by 26.3–27.6% amino acids (Figure 3C), which we speculate may be enough to influence vaccine candidates to produce different potencies. In general, the development of neutralizing antibody detection methods for different strains of MARV is an integral part of its vaccine development and an essential indicator of antibody–drug evaluation.

Pseudovirus is a classic alternative to natural viruses for antibody neutralizing activity evaluation, especially for various high-risk pathogens that require laboratory manipulation above the P2 level. The construction strategy of most pseudoviruses is replication-deficient to ensure low biosecurity risk. Pseudoviruses obtained by packaging can infect target cells but cannot replicate and proliferate in cells. Therefore, using such pseudoviruses to evaluate the neutralizing activity of antibodies requires frequent viral packaging. Each operation has potential factors that affect the packaging efficiency of pseudoviruses, such as plasmid purity and cell state. rS-MARV solves this dilemma because they are safe and reproducible. ATP, magnesium ions, and oxygen can catalyze the oxidation of luciferin to oxyluciferin. The oxidation process can produce bioluminescence at a wavelength of about 560 nm. A luminometer can measure bioluminescence. The bioluminescence system of luciferin and luciferase can detect the expression of the reporter gene. Pseudoviruses carrying luciferase have been developed into detection kits by various manufacturers and have become the “gold standard” for evaluating neutralizing antibodies (such as PSV016, SinoBiological; L02087A, GenScript; 1,001–001, and IBT BIOSERVICES). However, this pseudo viral system must lyse cells to determine luciferase activity in terms of neutralizing activity. The eGFP reporter gene eliminates these tedious steps and can be directly observed under a fluorescence microscope. Therefore, rS-MARV is more suitable for quickly and accurately determining the neutralizing activity of antibodies. The replicable pseudovirus carrying eGFP has better application prospects than the commercially available pseudovirus.

This study developed a replicable pseudovirus using RABV as a vector and carrying the eGFP gene to evaluate MARV neutralizing antibodies. rS-MARV showed high safety in safety evaluation, and all 3-day-old ICR suckling mice survived after intracranial injection. These viruses generally replicate in the mouse brain and exhibit mild pathological changes (Figures 6A,D). Therefore, they have the primary conditions to operate in the P2 laboratory, and at the same time, we use them to improve the specific operation links. To highlight the features of this approach, we found their respective advantages when comparing them with a lentivirus-based pseudovirus (rLV-MARV). 1. Cycle and cost: The rLV-MARV method has a more extended detection period and is more costly. This form of pseudovirus requires freshly rescued viral fluid prior to detection. During the experiment, we found that this pseudoviruses physical and chemical properties are unstable and are very sensitive to temperature and storage time. For example, after being stored at −80°C for one month, its infectivity was significantly reduced, and the reporter gene could not even be detected. Furthermore, the Luciferase gene relies on the bioluminescence. Gene expression often requires additional reagents to measure activity. Fresh virus solution also increases the cost of transfection reagents and extraction of endotoxin-free high-purity plasmids. 2. Application scenarios: rS-MARV is more suitable for accurate detection, and rLV-MARV is more suitable for large-scale primary screening. The rS-MARV method requires serial dilution of the sample, and the neutralization activity is judged according to the fluorescence in the well at different dilutions. That also makes it impossible to determine whether there is neutralizing activity after direct incubation of undiluted samples with the virus, which rLV-MARV can do. 3. Stability: The rS-MARV method requires 10^2^ TCID_50_, and the virus can be diluted in advance and stored at −80°C for flexible operation. However, rLV-MARV has certain limitations. Even with a new rLV-MARV virus solution, the RLU value of the virus control will be different. At the same time, we found no proportional relationship between the RLU value and the dilution factor. These problems may affect the final statistical results.

Comparison of neutralizing activity of MR191 using different methods showed the same results, namely, the neutralizing ability of MR191 to different strains was Angola>Musoke>Ravn in descending order. That is consistent with the neutralization results of MR191 against natural MARV reported by Andrew I. Flyak et al. Three 3 T3 cell lines expressing MARV GP were used to reflect the binding activity of MR191 to GP. Combined with the results of ELISA, it can be seen that the binding ability of MR191 to GP from strong to weak is as follows: Musoke>Ravn>Angola. Based on these results, we believe there is no equivalence between MARV antibodies’ neutralizing and binding activities. Moreover, it was also confirmed that the MARV GP protein expressed by murine 3 T3 cells could also produce an immune reaction with the human MR191 antibody.

In summary, we have developed three pseudoviruses that can serve as safe and convenient systems for studying MARV infection, evaluating vaccine efficacy, and developing therapeutic antibody drugs. The development of pseudoviruses for different strains will also greatly facilitate the development of pan-filovirus vaccines. There are currently no approved vaccines and antibody drugs against MARV, and most studies are also in preclinical stages. We also hope to make these pseudoviruses and related protocols available to vaccine and antibody developers to help them evaluate product candidates and advance the development of MARV prevention and treatment drugs. Furthermore, this pseudovirus assay also helps screen various potential use scenarios for characterizing MARV GP, including chemosynthetic drugs that inhibit MARV invasion into the host.

## Data Availability Statement

The original contributions presented in the study are included in the article/Supplementary Materials, and further inquiries can be directed to the corresponding authors.

## Ethics Statement

All the experimental mice in this study were kept following the “National Standards for Welfare of Laboratory Animals in China” (GB14925-2010). This study was approved by the Animal Welfare and Ethics Committee of the Changchun Veterinary Institute of the Chinese Academy of Agricultural Sciences.

## Author Contributions

XX, YZ, and FY formulate a hypothesis and design an experiment. JB, HW, HP, QH, QW, XW, ZW, and SW performed the experiments. JB, NF, and SY analyzed and discussed the data. JB wrote the manuscript. LG, MW, and HL provided the necessary experimental equipment. All authors contributed to the article and approved the submitted version.

## Funding

This work was supported by the National Key Research and Development Program of China (2021YFF0703600).

## Conflict of Interest

The authors declare that the research was conducted in the absence of any commercial or financial relationships that could be construed as a potential conflict of interest.

## Publisher’s Note

All claims expressed in this article are solely those of the authors and do not necessarily represent those of their affiliated organizations, or those of the publisher, the editors and the reviewers. Any product that may be evaluated in this article, or claim that may be made by its manufacturer, is not guaranteed or endorsed by the publisher.

## Supplementary Material

Supplementary Figure 1 Western blot analysis of different antibodies and pseudoviruses.

Supplementary Figure 2 Sequencing results of the fifth generation pseudovirus genome identified by RT-PCR. Colored sequences are the samples that were sequenced and correspond to the standard sequences.

Supplementary Figure 3 Sequencing results of the tenth generation pseudovirus genome identified by RT-PCR. Colored sequences are the samples that were sequenced and correspond to the standard sequences.

## References

[ref1] Abir M. H. Rahman T. Das A. Etu S. N. Nafiz I. H. Rakib A.. (2022). Pathogenicity and virulence of Marburg virus. Virulence 13, 609–633. doi: 10.1080/21505594.2022.2054760, PMID: 35363588 PMC8986239

[ref2] Barrette R. W. Xu L. Rowland J. M. McIntosh M. T. (2011). Current perspectives on the phylogeny of Filoviridae. Infect. Genet. Evol. 11, 1514–1519. doi: 10.1016/j.meegid.2011.06.017, PMID: 21742058 PMC7106080

[ref3] Bournazos S. DiLillo D. J. Ravetch J. V. (2015). The role of fc-FcgammaR interactions in IgG-mediated microbial neutralization. J. Exp. Med. 212, 1361–1369. doi: 10.1084/jem.20151267, PMID: 26282878 PMC4548051

[ref4] Bramble M. S. Hoff N. Gilchuk P. Mukadi P. Lu K. Doshi R. H.. (2018). Pan-Filovirus serum neutralizing antibodies in a subset of Congolese Ebolavirus infection survivors. J. Infect. Dis. 218, 1929–1936. doi: 10.1093/infdis/jiy453, PMID: 30107445 PMC6217721

[ref5] Cao Z. Jin H. Wong G. Zhang Y. Jiao C. Feng N.. (2021). The application of a safe neutralization assay for Ebola virus using Lentivirus-based Pseudotyped virus. Virol. Sin. 36, 1648–1651. doi: 10.1007/s12250-021-00405-8, PMID: 34152565 PMC8692649

[ref6] Cross R. W. Fenton K. A. Geisbert J. B. Ebihara H. Mire C. E. Geisbert T. W. (2015). Comparison of the pathogenesis of the Angola and Ravn strains of Marburg virus in the outbred Guinea pig model. J. Infect. Dis. 212, S258–S270. doi: 10.1093/infdis/jiv182, PMID: 26092858 PMC4564542

[ref7] Dulin N. Spanier A. Merino K. Hutter J. N. Waterman P. E. Lee C.. (2021). Systematic review of Marburg virus vaccine nonhuman primate studies and human clinical trials. Vaccine 39, 202–208. doi: 10.1016/j.vaccine.2020.11.042, PMID: 33309082

[ref8] Flyak A. I. Ilinykh P. A. Murin C. D. Garron T. Shen X. Fusco M. L.. (2015). Mechanism of human antibody-mediated neutralization of Marburg virus. Cell 160, 893–903. doi: 10.1016/j.cell.2015.01.031, PMID: 25723164 PMC4344968

[ref9] Guinea tMoHo (2021). Marburg virus disease – Guinea. 6 August 2021. World Health Organization. Available at: https://www.who.int/emergencies/disease-outbreak-news/item/2021-DON331

[ref10] Hashiguchi T. Fusco M. L. Bornholdt Z. A. Lee J. E. Flyak A. I. Matsuoka R.. (2015). Structural basis for Marburg virus neutralization by a cross-reactive human antibody. Cell 160, 904–912. doi: 10.1016/j.cell.2015.01.041, PMID: 25723165 PMC4344967

[ref11] Hevey M. Negley D. Pushko P. Smith J. Schmaljohn A. (1998). Marburg virus vaccines based upon alphavirus replicons protect guinea pigs and nonhuman primates. Virology 251, 28–37. doi: 10.1006/viro.1998.9367, PMID: 9813200

[ref12] Hevey M. Negley D. Staley A. Schmaljohn A. (2001). “Determination of vaccine components required for protecting cynomolgus macaques against genotypically divergent isolates of Marburg virus,” in *20th Annual Meeting of the American Society for Virology*; July 2001.

[ref13] Hume A. J. Muhlberger E. (2019). Distinct genome replication and transcription strategies within the growing Filovirus family. J. Mol. Biol. 431, 4290–4320. doi: 10.1016/j.jmb.2019.06.029, PMID: 31260690 PMC6879820

[ref14] Keck Z. Y. Enterlein S. G. Howell K. A. Vu H. Shulenin S. Warfield K. L.. (2016). Macaque monoclonal antibodies targeting novel conserved epitopes within Filovirus glycoprotein. J. Virol. 90, 279–291. doi: 10.1128/JVI.02172-15, PMID: 26468532 PMC4702572

[ref15] Keshwara R. Hagen K. R. Abreu-Mota T. Papaneri A. B. Liu D. Wirblich C.. (2019). A recombinant rabies virus expressing the Marburg virus glycoprotein is dependent upon antibody-mediated cellular cytotoxicity for protection against Marburg virus disease in a murine model. J. Virol. 93:e01865-18. doi: 10.1128/JVI.01865-18, PMID: 30567978 PMC6401435

[ref16] King L. B. Fusco M. L. Flyak A. I. Ilinykh P. A. Huang K. Gunn B.. (2018). The Marburgvirus-neutralizing human monoclonal antibody MR191 targets a conserved site to block virus receptor binding. Cell Host Microbe 23, 101–109. doi: 10.1016/j.chom.2017.12.003, PMID: 29324225 PMC5772738

[ref17] Klasse P. J. Sattentau Q. J. (2002). Occupancy and mechanism in antibody-mediated neutralization of animal viruses. J. Gen. Virol. 83, 2091–2108. doi: 10.1099/0022-1317-83-9-2091, PMID: 12185262

[ref18] Li Q. Liu Q. Huang W. Li X. Wang Y. (2018). Current status on the development of pseudoviruses for enveloped viruses. Rev. Med. Virol. 28:e1963. doi: 10.1002/rmv.1963, PMID: 29218769 PMC7169153

[ref19] Mahase E. (2021). Guinea reports West Africa's first ever case of Marburg virus disease. BMJ 374:n1988. doi: 10.1136/bmj.n1988, PMID: 34376410

[ref20] Mire C. E. Geisbert J. B. Borisevich V. Fenton K. A. Agans K. N. Flyak A. I.. (2017). Therapeutic treatment of Marburg and Ravn virus infection in nonhuman primates with a human monoclonal antibody. Sci. Transl. Med. 9:eaai8711. doi: 10.1126/scitranslmed.aai8711, PMID: 28381540 PMC5719873

[ref21] Muruato A. E. Fontes-Garfias C. R. Ren P. Garcia-Blanco M. A. Menachery V. D. Xie X.. (2020). A high-throughput neutralizing antibody assay for COVID-19 diagnosis and vaccine evaluation. Nat. Commun. 11:4059. doi: 10.1038/s41467-020-17892-0, PMID: 32792628 PMC7426916

[ref22] Nie J. Li Q. Wu J. Zhao C. Hao H. Liu H.. (2020). Establishment and validation of a pseudovirus neutralization assay for SARS-CoV-2. Emerg. Microbes Infect. 9, 680–686. doi: 10.1080/22221751.2020.1743767, PMID: 32207377 PMC7144318

[ref23] Nyakarahuka L. Kankya C. Krontveit R. Mayer B. Mwiine F. N. Lutwama J.. (2016). How severe and prevalent are Ebola and Marburg viruses? A systematic review and meta-analysis of the case fatality rates and seroprevalence. BMC Infect. Dis. 16:708. doi: 10.1186/s12879-016-2045-6, PMID: 27887599 PMC5124280

[ref24] Nyakarahuka L. Ojwang J. Tumusiime A. Balinandi S. Whitmer S. Kyazze S.. (2017). Isolated case of Marburg virus disease, Kampala, Uganda, 2014. Emerg. Infect. Dis. 23, 1001–1004. doi: 10.3201/eid2306.170047, PMID: 28518032 PMC5443453

[ref25] Park J. K. Xiao Y. Ramuta M. D. Rosas L. A. Fong S. Matthews A. M.. (2020). Pre-existing immunity to influenza virus hemagglutinin stalk might drive selection for antibody-escape mutant viruses in a human challenge model. Nat. Med. 26, 1240–1246. doi: 10.1038/s41591-020-0937-x, PMID: 32601336 PMC7450362

[ref26] Paweska J. T. Storm N. Markotter W. Di Paola N. Wiley M. R. Palacios G. (2020). Jansen van Vuren P. shedding of Marburg virus in naturally infected Egyptian Rousette bats, South Africa, 2017. Emerg. Infect. Dis. 26, 3051–3055. doi: 10.3201/eid2612.202108, PMID: 33219802 PMC7706944

[ref27] Rahim M. N. Wee E. G. He S. Audet J. Tierney K. Moyo N.. (2019). Complete protection of the BALB/c and C57BL/6J mice against Ebola and Marburg virus lethal challenges by pan-filovirus T-cell epigraph vaccine. PLoS Pathog. 15:e1007564. doi: 10.1371/journal.ppat.1007564, PMID: 30817809 PMC6394903

[ref28] Ramesh A. K. Parreno V. Schmidt P. J. Lei S. Zhong W. Jiang X.. (2020). Evaluation of the 50% infectious dose of human Norovirus Cin-2 in Gnotobiotic pigs: A comparison of classical and contemporary methods for endpoint estimation. Viruses 12:955. doi: 10.3390/v12090955, PMID: 32872283 PMC7552045

[ref29] Ristanovic E. S. Kokoskov N. S. Crozier I. Kuhn J. H. Gligic A. S. (2020). A Forgotten Episode of Marburg Virus Disease: Belgrade, Yugoslavia. Microbiol. Mol. Biol. Rev. 84:00095-19. doi: 10.1128/MMBR.00095-19 PMC7233485 32404328

[ref30] Salazar-Garcia M. Acosta-Contreras S. Rodriguez-Martinez G. Cruz-Rangel A. Flores-Alanis A. Patino-Lopez G.. (2021). Pseudotyped vesicular stomatitis virus-severe acute respiratory syndrome-Coronavirus-2 spike for the study of variants, vaccines, and therapeutics Against coronavirus disease 2019. Front. Microbiol. 12:817200. doi: 10.3389/fmicb.2021.817200, PMID: 35095820 PMC8795712

[ref31] Selvaraj S. A. Lee K. E. Harrell M. Ivanov I. Allegranzi B. (2018). Infection rates and risk factors for infection Among health workers During Ebola and Marburg virus outbreaks: A systematic review. J. Infect. Dis. 218:S679. doi: 10.1093/infdis/jiy435, PMID: 30202878 PMC6249600

[ref32] Siya A. Bazeyo W. Tuhebwe D. Tumwine G. Ezama A. Manirakiza L.. (2019). Lowland grazing and Marburg virus disease (MVD) outbreak in Kween district, eastern Uganda. BMC Public Health 19:136. doi: 10.1186/s12889-019-6477-y, PMID: 30704427 PMC6357374

[ref33] Swenson D. L. Warfield K. L. Negley D. L. Schmaljohn A. Aman M. J. Bavari S. (2005). Virus-like particles exhibit potential as a pan-filovirus vaccine for both Ebola and Marburg viral infections. Vaccine 23, 3033–3042. doi: 10.1016/j.vaccine.2004.11.070, PMID: 15811650

[ref34] Tohma K. Ford-Siltz L. A. Kendra J. A. Parra G. I. (2022). Dynamic immunodominance hierarchy of neutralizing antibody responses to evolving GII.4 noroviruses. Cell Rep. 39:110689. doi: 10.1016/j.celrep.2022.110689, PMID: 35417705

[ref35] Vial C. Whitaker A. Wilhelm J. Ovalle J. Perez R. Valdivieso F.. (2020). Comparison of VSV Pseudovirus and focus reduction neutralization assays for measurement of anti-Andes orthohantavirus neutralizing antibodies in patient samples. Front. Cell. Infect. Microbiol. 10:444. doi: 10.3389/fcimb.2020.00444, PMID: 33042854 PMC7527604

[ref36] Wang H. Jin H. Feng N. Zheng X. Li L. Qi Y.. (2015). Using rabies virus vaccine strain SRV9 as viral vector to express exogenous gene. Virus Genes 50, 299–302. doi: 10.1007/s11262-014-1160-y, PMID: 25724175

[ref37] Whitt M. A. (2010). Generation of VSV pseudotypes using recombinant DeltaG-VSV for studies on virus entry, identification of entry inhibitors, and immune responses to vaccines. J. Virol. Methods 169, 365–374. doi: 10.1016/j.jviromet.2010.08.006, PMID: 20709108 PMC2956192

[ref38] Xiang Q. Li L. Wu J. Tian M. Fu Y. (2022). Application of pseudovirus system in the development of vaccine, antiviral-drugs, and neutralizing antibodies. Microbiol. Res. 258:126993. doi: 10.1016/j.micres.2022.126993, PMID: 35240544 PMC8848573

[ref39] Zamoiskii E. A. (1956). Evaluation of Reed-Muench method in determination of activity of biological preparations. Zh Mikrobiol. Epidemiol. Immunobiol. 27, 77–83. 13325889

