# Supplementary figures and images for: A Novel and Secure Pseudovirus Reporter System Based Assay for Neutralizing and Enhancing Antibody Assay Against Marburg Virus
Source: Front Microbiol. 2022 Jun 9;13:927122. doi: 10.3389/fmicb.2022.927122 (PMC9224600; doi:10.3389/fmicb.2022.927122)

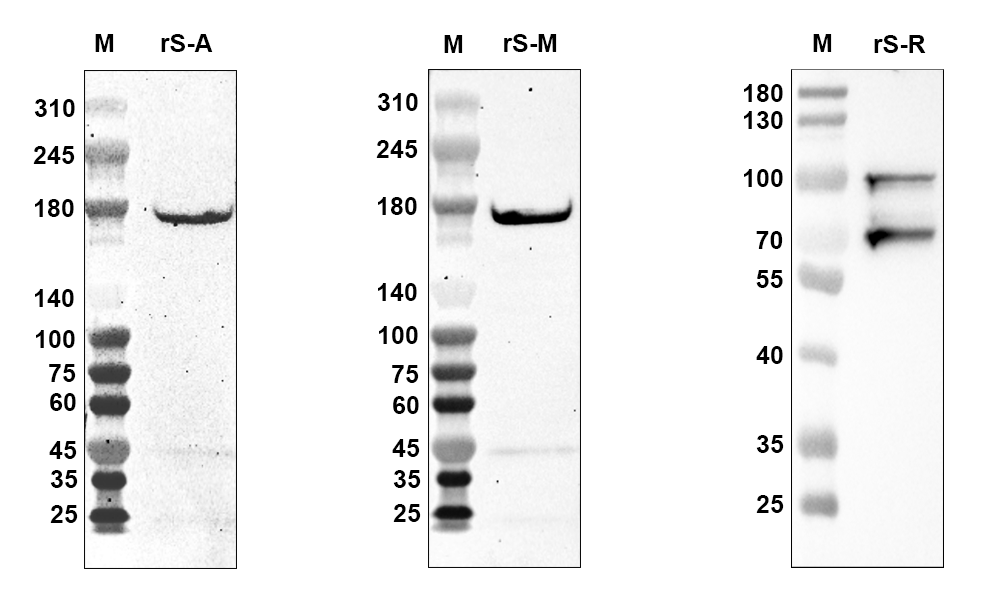

Supplement: Supplementary Figure 1 — Western blot analysis of different antibodies and pseudoviruses. [file Image_1.TIF]

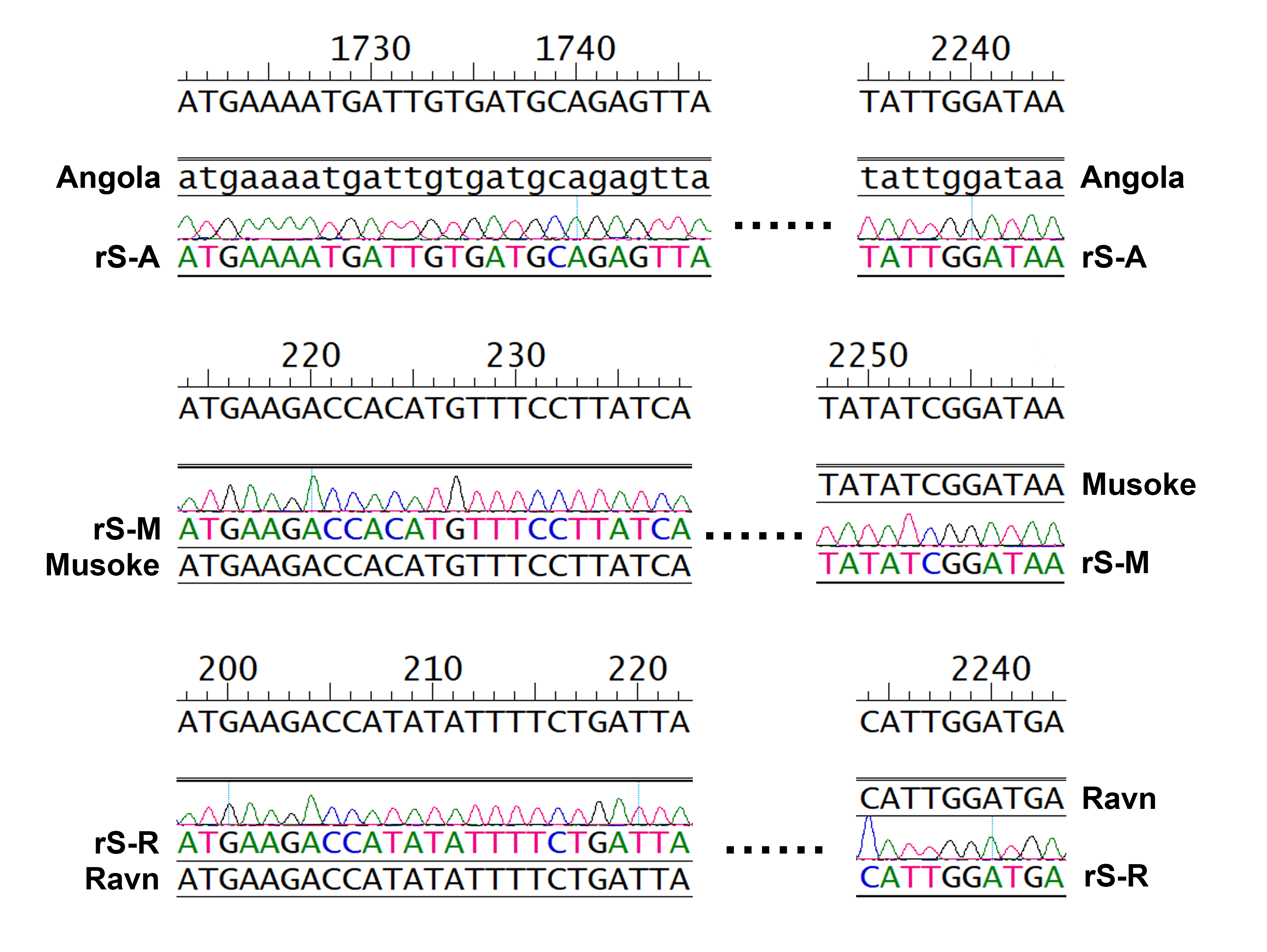

Supplement: Supplementary Figure 2 — Sequencing results of the fifth generation pseudovirus genome identified by RT-PCR. Colored sequences are the samples that were sequenced and correspond to the standard sequences. [file Image_2.TIF]

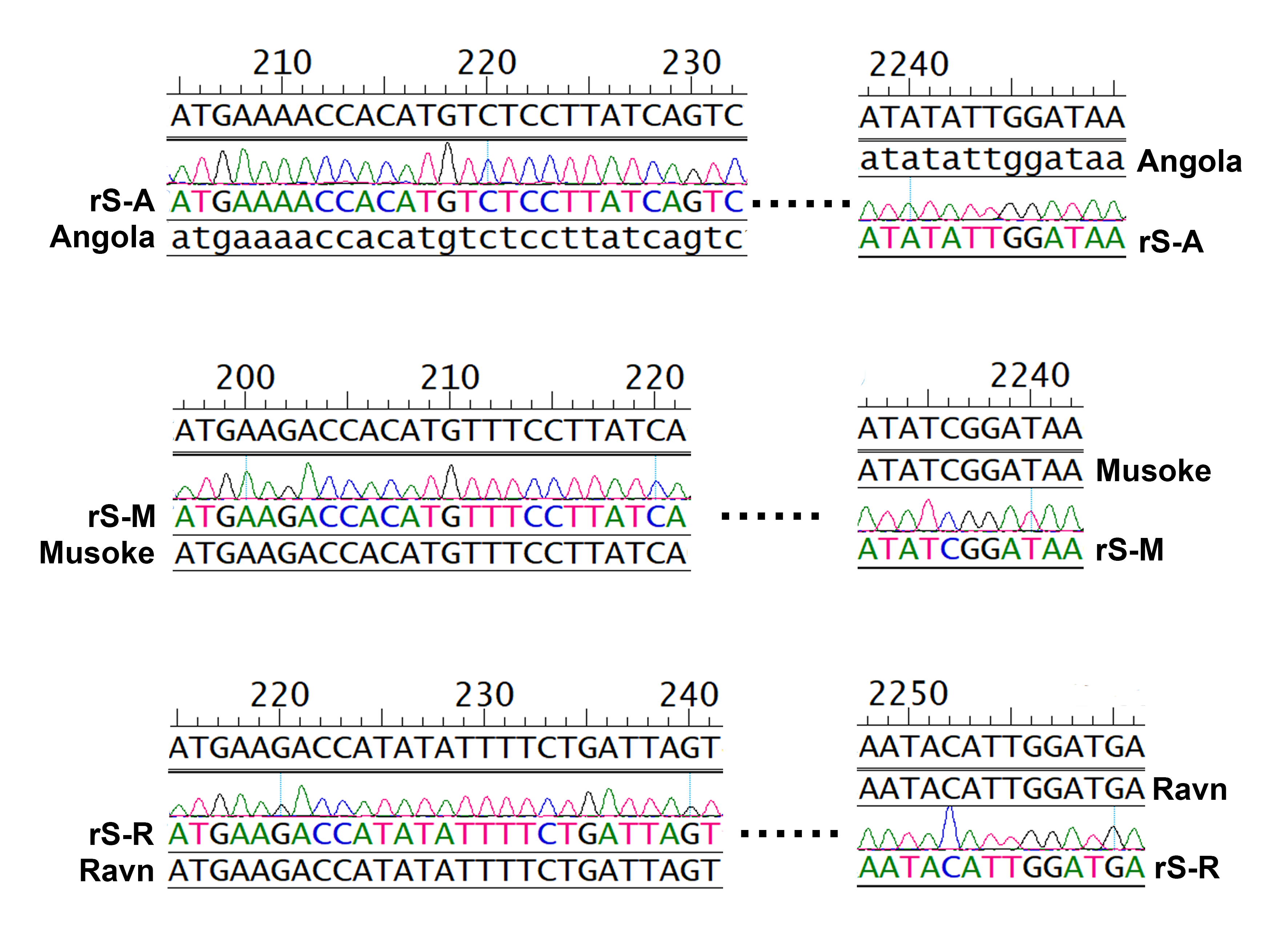

Supplement: Supplementary Figure 3 — Sequencing results of the tenth generation pseudovirus genome identified by RT-PCR. Colored sequences are the samples that were sequenced and correspond to the standard sequences. [file Image_3.TIF]
