# Supplementary material for: A Novel and Secure Pseudovirus Reporter System Based Assay for Neutralizing and Enhancing Antibody Assay Against Marburg Virus
Source: Front Microbiol. 2022 Jun 9;13:927122. doi: 10.3389/fmicb.2022.927122 (PMC9224600; doi:10.3389/fmicb.2022.927122)
Supplement: Supplementary file 4 [file Table_1.DOCX]

**1. MR191 sequence**

**> NO.2_Target_MR191-VH (467bp)**

GAATTCGCCACCATGTACCGGATGCAGCTGCTGAGCTGCATCGCCCTGAGCCTGGCCCTGGTGACAAACAGCCAGCTGCAGCTGCAGGAGAGCGGCCCCGGCCTGGTGAAGCCCAGCGAGACACTGAGCCTGAGCTGCACAGTGAGCGGGGTGAGCATCAGCGATAACAGCTACTACTGGGGGTGGATCCGGCAGCCCCCAGGGAAGGGCCTGGAGTGGATCGGCACCATCAGCTACAGCGGCAACACCTACTACAACCCCAGCCTCAAGAGCCGGGTGAGCATCAGCGGGGATACCAGCAAGCACCAGCTGAGCCTGAAGGTGAGCAGCGTGACAGCCGCCGACACCGCCGTGTACTACTGCGCCAGGCAGAGGATCGTGAGCGGCTTCGTGGAGTGGCTGAGCAAGTTCGATTACTGGGGCCAGGGGACACTGGTGACAGTGAGCTCCGCCAGCACCAAGGGCCC

**> NO.2_Target_MR191-VL (732bp)**

GAATTCGCCACCATGTACCGGATGCAGCTGCTGAGCTGCATCGCCCTGAGCCTGGCCCTGGTGACAAACAGCCAGAGCGTGCTGACACAGCCACCTAGCGTGAGCGGCGCCCCTGGCCAGCGGGTGACCATCAGCTGCACCGGGAGCAGCAGCAACATCGGCGCCGGGTTCGACGTGCACTGGTACCAGCAGCTGCCCGGCACCGCCCCCAAGCTGCTGATCTACGATAACAATAACAGGCCCAGCGGCGTGCCCGATAGGTTTAGCGGGAGCAAGAGCGGGACAAGCGCCAGCCTGGCCATCACAGGGCTGCAGGCCGAGGACGAGGCCGACTACTACTGCCAGAGCTACGACACCAGCCTGAGCGGCCCCGTGGTGTTCGGGGGCGGGACAAAGCTGACAGTGCTGCAGCCAAAGGCCGCCCCAAGCGTGACCCTGTTCCCACCCTCCAGCGAGGAACTGCAGGCTAACAAAGCCACACTGGTGTGTCTGATCTCCGACTTCTATCCTGGCGCCGTGACCGTGGCCTGGAAGGCCGACAGCAGCCCCATCAAAGCCGGCGTGGAGACAACCACACCCAGCAAGCAGTCCAATAACAAGTACGCCGCCAGCAGTTACCTGTCTCTGACACCCGAGCAGTGGAAGTCACATAGGAGCTACTCTTGTCAGGTGACCCATGAAGGCAGCACAGTGGAGAAGACAGTGGCCCCCACAGAGTGCAGCTGAAAGCTT
